# Overall Survival Benefits for Combining Targeted Therapy as Second-Line Treatment for Advanced Non-Small-Cell-Lung Cancer: A Meta-Analysis of Published Data

**DOI:** 10.1371/journal.pone.0055637

**Published:** 2013-02-08

**Authors:** Wei-Xiang Qi, Qiong Wang, Yan-Ling Jiang, Yuan-Jue Sun, Li-na Tang, Ai-na He, Da-liu Min, Feng Lin, Zan Shen, Yang Yao

**Affiliations:** 1 Department of Oncology, the Sixth People’s Hospital, Shanghai Jiao Tong University, Shanghai, China; 2 Department of Oncology, The Kunming Medical University, Kunming, Yunnan, China; University of Nebraska Medical Center, United States of America

## Abstract

**Background:**

Combining targeted therapy has been extensively investigated in previously treated advanced non-small-cell lung cancer (NSCLC), but it is still unclear whether combining targeted therapy might offer any benefits against standard monotherapy with erlotinib. We thus performed a meta-analysis of randomized controlled trials to compare the efficacy and safety of combining targeted therapy versus erlotinib alone as second-line treatment for advanced NSCLC.

**Methods:**

Several databases were searched, including Pubmed, Embase and Cochrane databases. The endpoints were overall survival (OS), progression-free survival (PFS), overall response rate (ORR) and grade 3 or 4 adverse event (AEs). The pooled hazard ratio (HR) or odds ratio (OR), and 95% confidence intervals (CI) were calculated employing fixed- or random-effects models depending on the heterogeneity of the included trials.

**Results:**

Eight eligible trials involved 2417 patients were ultimately identified. The intention to treatment (ITT) analysis demonstrated that combining targeted therapy significantly improved OS (HR 0.90, 95%CI: 0.82–0.99, *p* = 0.024), PFS (HR 0.83, 95%CI: 0.72–0.97, *p* = 0.018), and ORR (OR 1.35, 95%CI 1.01–1.80, *P* = 0.04). Sub-group analysis based on phases of trials, EGFR-status and KRAS status also showed that there was a tendency to improve PFS and OS in combining targeted therapy, except that PFS for patients with EGFR-mutation or wild type KRAS favored erlotinib monotherapy. Additionally, more incidence of grade 3 or 4 rash, fatigue and hypertension were observed in combining targeted therapy.

**Conclusions:**

With the available evidence, combining targeted therapy seems superior over erlotinib monotherapy as second-line treatment for advanced NSCLC. More studies are still needed to identify patients who will most likely benefit from the appropriate combining targeted therapy.

## Introduction

Lung cancer remains the leading cause of malignancy-related mortality worldwide, with over one million deaths worldwide each year [1]. Non-small-cell lung cancer (NSCLC) accounts for more than 80% of lung cancers and most patients present with locally advanced or metastatic disease at the time of diagnosis. Therefore, most patients will face the option of palliative chemotherapy, with prolonging patient’s survival and quality of life as the primary end-points. To date, platinum-based doublet chemotherapy remains the cornerstone of treatment in advanced NSCLC [2], [3]. However, although most patients initially achieve clinical remission or disease stabilization with first-line therapy, nearly all experience disease progression and require second-line therapy.

More recently, the achievement of a therapeutic plateau with chemotherapy, as well as the increased knowledge of tumor biology and the molecular pathways involved in cancer cells proliferation, have represented the main rationale for developing targeted agents that specifically block dysregulated signaling pathways and the metabolic processes contributing to the acquisition of a cancer phenotype [4], [5], [6], [7], [8]. Erlotinib, a small molecule inhibitor of the intracellular tyrosine kinase of endothelial growth factor receptor, has been approved as second-line therapy for advanced NSCLC in many countries [9], [10], [11], [12], [13]. However, given the heterogeneity of this tumor type and potential crosstalk between key signaling pathways, the efficacy of erlotinib monotherapy as second-line treatment for advanced NSCLC is limited, including low response rate (8.9%), brief duration of disease control and minimal survival advantage [9], [11].

One potential strategy to offer additional clinical benefits for advanced NSCLC is to inhibit multiple key signaling pathways by using multitargeted agents or a combination of targeted agents. Indeed, synergistic antitumor activity achieved by combining targeted agents has been observed in preclinical and clinical studies [3], [14], [15], [16]. Furthermore, introducing combination therapy early in the course of a disease could prevent the emergence of drug resistance [17], [18], [19], [20]. As a result, several trials have been conducted in recent years to determine the clinical benefits gained from a number of combined inhibition strategies in this setting, but most of these trials are characterized by a small sample size, with inadequately statistical power to exclude clinically relevant differences in efficacy. We thus perform this meta-analysis to compare the efficacy and safety of combining targeted therapy versus erlotinib alone as second-line treatment for advanced NSCLC.

## Methods

### Search Strategy

We searched PubMed (up to May 2012), Embase (1980 to May 2012), and the Cochrane Register of Controlled Trials using various combinations of different terms “advanced”, “metastatic” “non-small-cell lung cancer”, “second-line”, “erlotinib”, “targeted therapy”, “previously treated”, “randomized” and “tarceva” (see Search strategy S1). We also looked at posters from the annual meetings of the European Society of Medical Oncology (ESMO) and the American Society of Medical Oncology (ASCO) in the past 10 years. Moreover, we searched the Clinical Trials.gov (http://www.ClinicalTrials.gov) Web sites for information on registered RCTs. The search was limited to clinical studies in English language, and reference lists from relevant primary studies and review articles were also examined to find additional publications.

### Study Selection

The relevant clinical trials were manually selected carefully based on the following criteria: (1) trails comparing combining targeted therapy with erlotinib alone or erlotinib plus placebo; (2) patients with pathologically confirmed of advanced NSCLC and previously treated; (3) prospective phase II and III randomized controlled trials (RCTs); (4) The included study had sufficient data for extraction. Trials investigating immunotherapy or neoadjuvant or perioperative targeted therapy was excluded. Likewise, trials evaluating targeted agents plus chemotherapy therapy were not in the scope of our research. If multiple publications of the same trial were retrieved or if there was a case mix between publications, only the most recent publication (and the most informative) was included.

### Data Extraction

Data extraction and quality assessment were conducted independently by two reviewers using a standardized approach. Disagreements were adjudicated by a third reviewer after referring to the original articles. The following information was extracted from each article: (1). Basic information from papers such as, year of publication, phase of trials, and author name. (2).Characteristics of patients such as: median age, percent of female patients, EGFR mutation, and history of smoking. (3). Information of study designation such as: sample size per-group, study design, randomization scheme, inclusion criteria, and type of end point used. (4). Information of treatment such as: treatment regimens, median overall survival (OS), progression-free survival (PFS), 1-year survival rate (1-year SR), overall response rate (ORR), adverse events (AEs) and so on. Available information was extracted and recorded to a data collection form and entered into electronic database.

### Quality Assessment

An open assessment of the trials was performed using the methods reported by Jadad and colleagues [21], which assessed the trials according to the following three questions: (1)whether reported an appropriate randomization method (0–2 scores); (2) whether reported an appropriate blinding method (0–2 scores); (3) whether reported withdrawals and dropouts (0–1 scores). The quality scale ranged from 0 to 5 points, with a low-quality report receiving a score of 2 or less and a high-quality report receiving a score of at least 3.

### Data Analysis

The analysis was undertaken on an intention-to-treat basis: patients were analyzed according to treatment allocated, irrespective of whether they received that treatment. The outcomes used were (1) OS, defined as the time from random assignment to death from any cause, censoring patients who had not died at the date last known alive; (2) PFS, defined as the time from random assignment to first documented progression or death on study due to any cause, whichever occurred first; and (3) ORR, defined as the sum of partial and complete response rates according to the Response Evaluation Criteria in Solid Tumors [22]. For time-to-event data, the log hazard ratio (HRs) and their variances were estimated using the methods proposed by Parmar et al [23] when CIs of HRs were not reported. Otherwise, median survival time, events in each arm, and p values of the log-rank or Cox proportional hazard regression model were used to estimate log HRs and their variances. The summary HRs and their 95%CI were estimated using a general variance-based method. The AEs of treatments were analyzed as drug-related grades 3 or greater toxicity according to the National Cancer Institute common toxicity criteria (NCI-CTC) version 2 or 3 [24]. Estimates of the treatment effects and toxicity were obtained from the number of events reported in each arm and combined using Mantel-Haenszel methods [25].Between-study heterogeneity was estimated using the χ^2^-based Q statistic [26]. Heterogeneity was considered statistically significant when *P*_heterogeneity_ <0.05 or *I* ^2^>50%. If heterogeneity existed, data was analyzed using a random effects models (the DerSimonian and Laird method), as they give a more appropriate estimate of the average treatment effect in such trials, and usually yield wider CIs, thereby resulting in a more conservative statistical claim. In the absence of heterogeneity, a fixed effects model was used (the Mantel Haenszel methods). Sub-group analyses were also performed according to phases of trials, EGFR-status, and KRAS-status. A statistical test with a *p*-value less than 0.05 was considered significant. HR>1 reflected more deaths or progression in combining targeted agents therapy, and OR>1 indicated more toxicities and overall response rate in combining targeted agents therapy; and vice versa, HR<1 reflected less deaths or progression in combining targeted agents therapy, and OR<1 indicated less toxicities and overall response rate in combining targeted agents therapy. The presence of publication bias was evaluated by using the Begg and Egger tests [27], [28]. All p-values were two-sided. All CIs had a two-sided probability coverage of 95%. Statistical analysis of the overall hazard ratio (HR) for OS and PFS, the odds ratio (OR) for ORR and grade 3 or 4 AEs was calculated using Stata version 12.0 software (Stata Corporation, College Station, Texas, USA). Power calculation was carried out with the power and sample size calculation software [29] (PS version 3.0).

## Results

### Quantity and Quality of Evidence

**Figure 1:**
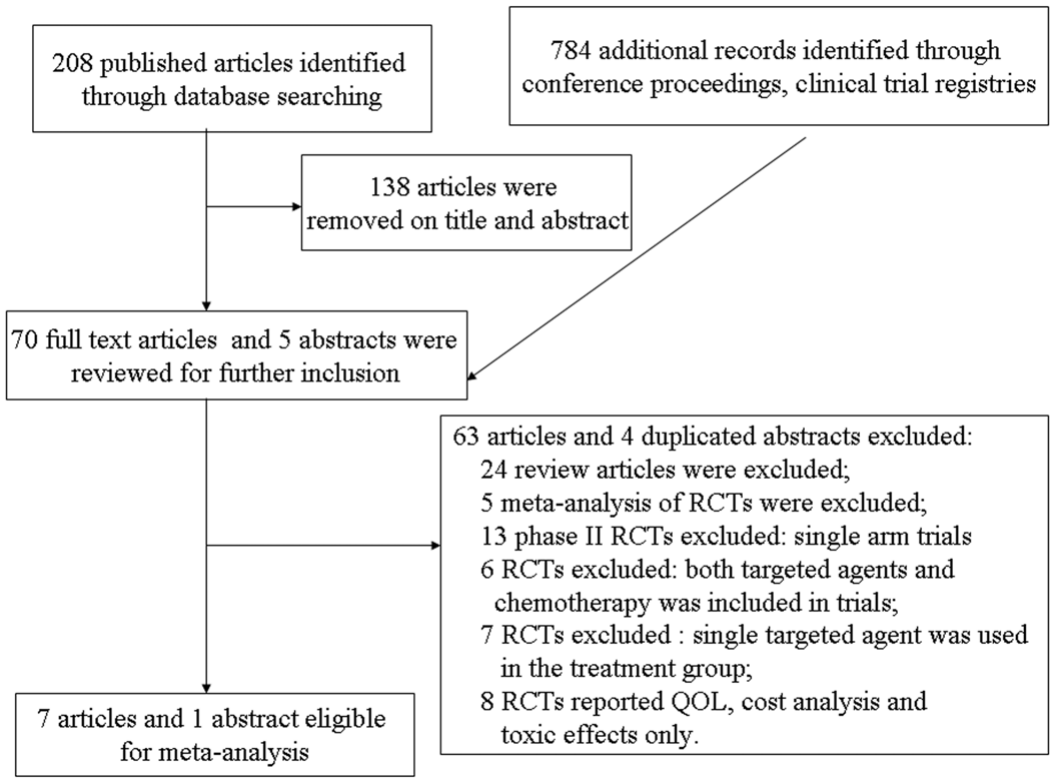
Flow chart of trial selection process.

The flow chart of our study was shown in figure 1. A total of 208 studies were retrieved electronically, 138 articles were removed on title and abstract, full-text copies of the remaining 70 citations were obtained and were evaluated in more detail. Of these, 63 articles were excluded for the following reasons: 5 citations were meta-analysis of RCTs; 24 citations were review articles; 13 trials were single arm phase II trials; 6 trials were RCTs, but both targeted agents and chemotherapy was included in trials; 7 trials were RCTs, but single targeted agent was used in the treatment group; 8 RCTs reported quality of life, cost analysis and toxic effects only. The remaining seven trials were included in the review. And one additional conference abstract was located as a result of hand searching. Finally, a total of 8 publications were therefore included in the review; these related to 7 clinical trials reported in the full-text publications [30], [31], [32], [33], [34], [35], [36] and 1 conference abstract [37]. The total number of randomized patients in these trials was 2417, with 1267 in the combining targeted agents arm and 1150 in the erlotinib alone arm. Six of 8 included trials were placebo-controlled double-blinded trial [31], [32], [33], [34], [35], [36], and two were large, phase III, multi-centre, randomized clinical studies [31], [34]. Characteristics of these eligible trials were given in table 1 and table 2. And six trials had Jadad scores of 5, which mentioned the concealment of allocation clearly in the randomization process, and provided the number of patients who withdrew from the trials. Another two trials, did not mention the blinding of allocation clearly in the randomization process, thus had Jadad scores of 3. We performed this meta-analysis in accordance with the guidelines of the Preferred Reporting Items for Systematic Review and Meta-analyses (PRISMA) statement [38] (see Checklist S1).

**Table 1:** overview of studies in the pooled analysis (N = 2417).

Study/year	Phase	Primary endpoint	Treatment regimen	No.of patients	CR+PR (%)	PFS, mo	OS, mo	1- Year SR (%)	Jadad score
**Lynch T.J.et al 2009**	II	ORR	Erlotinib/Bortezomib	25	9	1.3	8.5	40	3
			Erlotinib	25	16	2.7	7.3	30	
**Bennouna J. et al 2010**	II	NR	Erlotinib/Everolimus	66	12.1	2.9	NR	NR	3
			Erlotinib	67	10.4	2.0	NR	NR	
**HerBst, Roy S. et al** **2011**	III	OS	Erlotinib/bevacizumab	319	13	3.4	9.3	42.1	5
			Erlotinib/placebo	317	6	1.7	9.2	40.7	
**Sequist L.V. et al. 2011**	II	PFS	Erlotinib/tivantinib	84	10	3.8	8.5	NR	5
			Erlotinib/placebo	83	7	2.3	6.9	NR	
**Spigel D.R. et al. 2011**	II	ORR and PFS	Erlotinib/sorafenib	112	8	3.38	7.62	NR	5
			Erlotinib/placebo	56	11	1.94	7.23	NR	
**Ramalingam S.S.** **et al. 2011**	II	PFS	Erlotinib/R1507(IGF-1R)weekly	57	8.8	1.6	8.1	NR	5
			Erlotinib/R1507(IGF-1R)Q 3 weekly	57	7	2.7	12.1	NR	
			Erlotinib/placebo	57	8.8	1.5	8.1	NR	
**Scagliotti G.V. et al.** **2011**	III	OS	Erlotinib/sunitinib	480	10.6	3.6	9.0	NR	5
			Erlotinib/placebo	480	6.9	2.0	8.5	NR	
**Witta S.E.et al. 2012**	II	OS	Erlotinib/Entinostat	67	3.0	1.97	8.9	NR	5
			Erlotinib/placebo	65	9.2	1.88	6.7	NR	

Abbreviations: OS: overall survival; ORR: overall response rate; PFS: progression-free survival; CR: complete response; PR: partial response; 1-year SR: 1-year survival rate; NR: not reported.

**Table 2:** Characteristics of patients in the pooled analysis (N = 2417).

Study/year	Treatment arm	No.ofpatients	FemaleSex (%)	Medianage, y	History of smoking,%	KRAS mutation,n (%)	EGFR mutation,n (%)
**Lynch T.J. et al. 2009**	Combination	25	56	62	84	NR	NR
	Single	25	48	64	80	NR	NR
**Bennouna J. et al. 2010**	Combination	66	NR	59	80	NR	NR
	Single	67	NR	60	82	NR	NR
**HerBst, Roy S. et al. 2011**	Combination	319	46	64.8	89	48 (25)	33(32)
	Single	317	46	65	90	38 (21)	43(42)
**Sequist L.V. et al. 2011**	Combination	84	39	64	80	10 (17)	38(52)
	Single	83	41	62	78	5 (10)	59 (40)
**Spigel D.R. et al. 2011**	Combination	112	44	65	NR	5 (4.5)	22(19.6)
	Single	56	53	65	NR	6(10.7)	14(25)
**Ramalingam S.S. et al. 2011**	Combination(weekly)	57	32	63	86	16 (27)	NR
	Combination (every 3 weekly)	57	33	62	91	12(36)	NR
	Single	57	35	62	84	8 (19)	NR
**Scagliotti G.V. et al 2011**	Combination	480	38.1	61	80	NR	28(5.8)
	Single	480	40.8	61	81.3	NR	30(6.3)
**Witta S.E.et al. 2012**	Combination	67	42	66	84	4(9)	18(60)
	Single	65	34	67	83	7(21)	11(38)

### Pooled Analysis Results

**Figure 2:**
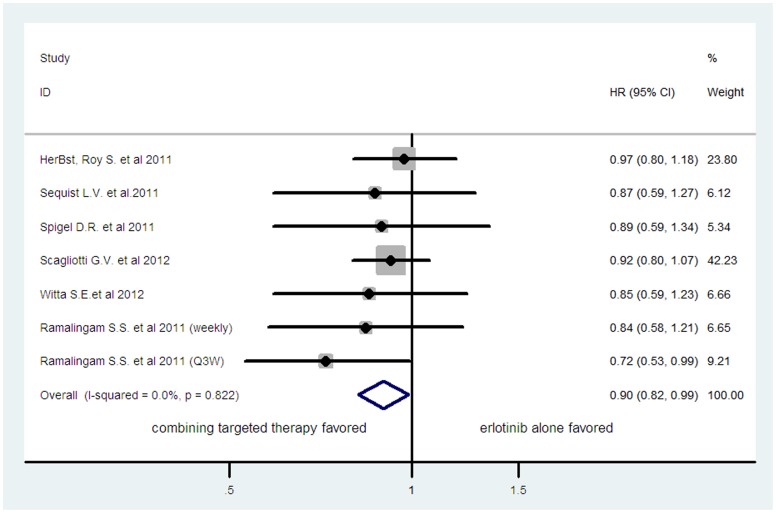
Comparison of OS between combining targeted therapy and erlotinib alone.

**Figure 3:**
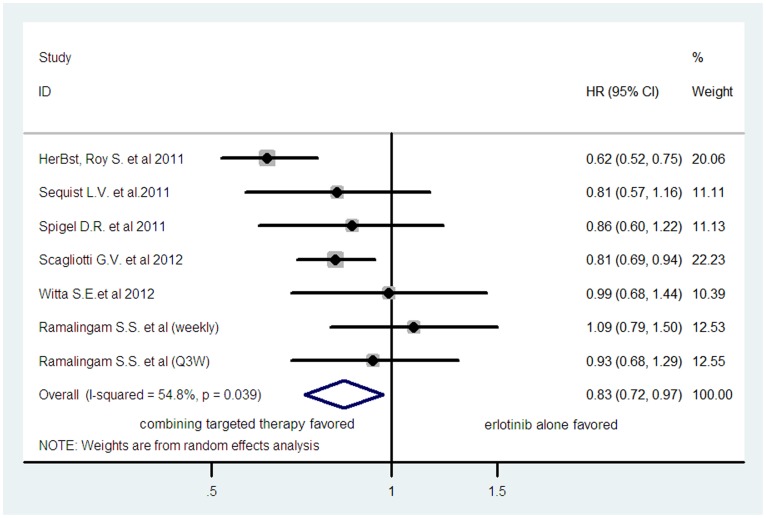
Comparison of PFS between combining targeted therapy and erlotinib alone.

**Figure 4:**
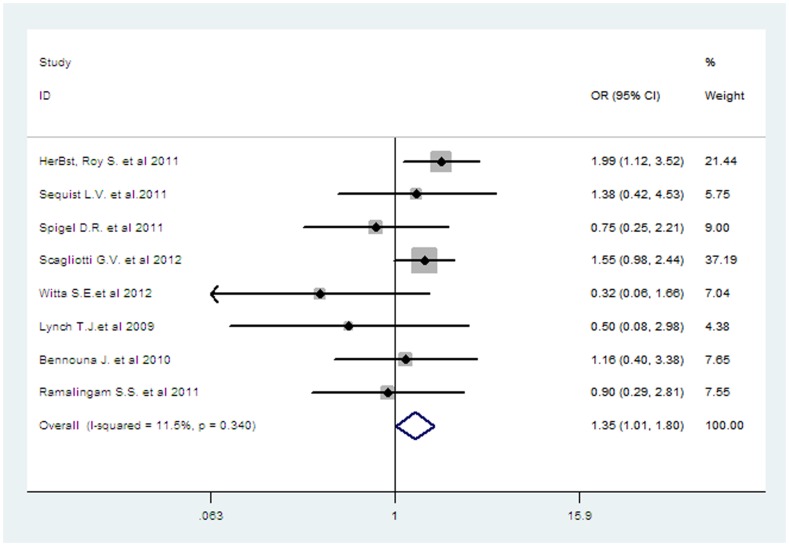
Comparison of ORR between combining targeted therapy and erlotinib alone.

Six of the 8 trials reported OS data [31], [32], [33], [34], [35], [36]. One trial was a three-arm study consisting of one control arm plus two experimental arms [36], thus there were two comparisons, the first between the control and first experimental arm and the second between the same control and second experimental arm. As a result, the total number of comparison was seven. The pooled hazard ratio for OS showed that there was a significant improvement in overall survival for combining targeted therapy with HR of 0.90 (95%CI: 0.82–0.99, *p* = 0.024; fixed-effect model) (figure 2), there was no significant heterogeneity between studies (*I* ^2^ = 0%, *p* = 0.822). Six trials reported PFS data [31], [32], [33], [34], [35], [36]. As one trial was a three-arm study [36], the number of comparison was seven. The pooled hazard ratio for PFS demonstrated that combining targeted therapy significantly improve PFS giving HR 0.83 (95%CI: 0.72–0.97, *p* = 0.018, figure 3), compared with erlotinib alone. There was significant heterogeneity between trials (*I* ^2^ = 54.8%, *p* = 0.039), and the pooled HR for PFS was performed by using random-effects model. All eight trials reported ORR data, and the pooled OR for overall response rate showed that there was a significant improvement for combining targeted therapy with OR 1.35 (95%CI 1.01–1.80, *P* = 0.04, figure 4).There was no significant heterogeneity between the trials (*I* ^2^ = 10.5%, *p* = 0.349), and the pooled RR for overall response was performed using fixed-effects model. Sub-group analysis could help us discover potential information of what the clinicians were interested in. Therefore, we studied some factors which might be related with survival between the two groups. Finally, phases of trials, EGFR-status and KRAS status were considered as the sub-group analysis factors. Overall, there was a tendency to improve PFS and OS in combining targeted therapy, except that PFS for patients with EGFR-mutation or KRAS with wild type favored erlotinib monotherapy (table 3). However, because of a small number of patients with EGFR-status and KRAS-status reported in these trials, it should be careful when interpreting these results. Totally, there are only 283 patients with EGFR mutation and 159 patients with KRAS mutation were included in our meta-analysis (table 2). In this light, more trials were still needed to identify molecular biomarkers that are predictive of efficacy. Pooled analysis of reported grades 3 and 4 adverse events (AEs) of interest was also performed used Mantel-Haenszel method. There were more incidences of grade 3 or 4 rash (OR1.34, 95%CI: 1.04–1.73, *p* = 0.023), fatigue (OR1.76, 95%CI: 1.18–2.64, p = 0.006), and hypertension (OR3.84, 95%CI: 1.35–10.89, p = 0.011) in combining targeted therapy. With regard to the risk of grade 3 or 4 anemia (OR1.25; 95%CI: 0.54–2.89, *p* = 0.602), and diarrhea (OR1.83, 95%CI: 0.63–5.34, *P* = 0.266), equivalent frequencies were found between the two groups (table 4). Begg’s funnel plot and Egger’s test were performed to assess the publication bias of literatures. The shapes of the funnel plots did not reveal any evidence of obvious asymmetry (*p* = 0.881 for OS, *p* = 0.548 for PFS, *p* = 0.108 for ORR, respectively). Then, Egger’s test was used to provide statistical evidence of funnel plot symmetry. The results also showed no evidence of publication bias (*p* = 0.162 for OS and *p* = 0.171 for PFS, respectively) except for ORR (*p* = 0.015).

**Table 3:** Sub-group analysis based on study characteristics.

Sub-group	No. of studies for PFS	HR (95%CI)	No. of studies for OS	OS (95%CI)
**Phases**				
** Phase II**	4 ^[28]^, [29], [31], ^[32]^	0.94 (0.80–1.09)	4 [^28]^, [29], [31], [32]	0.82 (0.70–0.97)
** Phase III**	2 [^27]^, [30]	0.71 (0.55–0.92)	2 [^27]^, [30]	0.94 (0.84–1.06)
** EGFR-status**				
** Wild type**	3 ^[^28]^, [29], [30]^	0.65 (0.42–0.88)	5 ^[^27]^, [28], [29], [30], [31]^	0.92 (0.75–1.12)
** Mutation**	2 [^28]^, [30]	1.20 (0.41–1.97)	3 ^[^27]^, [30], [31]^	0.91 (0.40–1.43)
**KRAS status**				
** Wild type**	1 ^[^28^]^	1.01 (0.63–1.60)	1 ^[^32^]^	0.71 (0.43–1.18 )
** Mutation**	1 [^28^]	0.18 (0.05–0.70)	2 [^28]^, [32]	0.37 (0.12–1.09)

**Table 4:** Comparison of grade 3 or 4 toxicities between combined targeted therapy and erlotinib alone.

Grade 3–4 Toxicity	Trials	Combined targetedtherapy	Single erlotinib	Heterogeneity		OR(95%CI)	*P* value
				*P* value	*I* ^2^		
Anemia	3	15/263	9/204	0.43	0	1.25(0.54–2.89)	0.602
Diarrhea	6	105/834	28/776	0.01	66.9%	1.83(0.63–5.34)	0.266
Rash	7	166/1201	115/1083	0.12	42.6%	1.34(1.04–1.73)	0.023
Fatigue	5	80/857	38/741	0.60	0	1.76(1.18–2.64)	0.006
Hypertension	2	19/431	4/373	0.90	0	3.84(1.35–10.89)	0.011

## Discussion

After progression following first-line treatment, many advanced NSCLC patients still have a good performance status and could be considered for further treatments. Until now, monotherapy with erlotinib is still the standard second-line treatment for advanced NSCLC. However, favorably clinical and preclinical data as well as sound biological reasons suggest that the next wave of new treatments for NSCLC will involve multitargeted molecular approaches into therapy. In addition, two recent systematic reviews about targeted therapy also found that combined inhibition of multiple signaling pathways could confer additive or synergistic antitumor effects and increase clinical benefit in patients with advanced NSCLC [3], [17]. Our meta-analysis combined 2417 patients from 8 randomized controlled trials so that treatment effect could be evaluated with greater statistical power. With the present sample size, we had a power of 85.5% to reject the null hypothesis that combining targeted therapy was inferior to erlotinib monotherapy as second-line therapy for advanced NSCLC, or a possibility of βerror of 14.5%. As far as we known, our study, for the first time, demonstrated a survival benefit of combining targeted therapy over monotherapy with erlotinib as second-line treatment for advanced NSCLC in terms of OS, PFS and ORR, and sub-group analysis based on phases of trials, EGFR-status and KRAS status also showed that there was a tendency to improve PFS and OS in combining targeted therapy, except that PFS for patients with EGFR-mutation or wild type KRAS favored erlotinib monotherapy. Overall, these encouraging data suggested that combining targeted therapy was a promising treatment strategy for advanced NSCLC. However, it should be note the fact that all of the trials, including 2 phase III trials, did not demonstrate overall survival benefits from combining therapy, although significant improvement in PFS and ORR had been observed in several trials. One possible explanation for this might be a relatively small number of patients included in each trial; thus these trials had no enough statistical power to evaluate the treatment effect of combining targeted therapy. This notion was supported by our meta-analysis results, which combined 2417 patients from 8 randomized controlled trials and showed a significant improvement in OS for combining therapy. Furthermore, because NSCLC was a heterogeneous disease, the magnitude of negative studies highlighted the fact these treatments were not “one size fits all”. In this light, a negative study might be more a reflection of an unselected patient population rather than disproof of a certain principle. As a result, it was of critical importance even in negative trials to identify molecular signatures that were predictive of response and to have information flow from bench to bedside and back.

Previous researches had demonstrated that geographic origin was an important factor influencing survival benefit from EGFR-TKIs monotherapy [39], [40], but all included trials in this study were conducted in Western countries. Therefore, whether Asian patients could gain survival benefits from combining targeted therapy was still unknown. In addition, we also found that the characters that well known to affect the efficacy and survival to EGFR-TKIs therapy, such as percentage of female patients, never smokers, and EGFR-mutation [11], [41], [42] (table 2), were not substantially different between unselected patients receiving combining targeted therapy and receiving single agent erlotinib in this study except for the most recent trial conducted by Scagliotti G V. et al [34].Though percentage of patients with EGFR mutation in this trial (6%) was lower than that in the other included trials (range from 19.6% to 50%), the median OS and PFS for patients in this study were comparable to those in other trials, which suggested that EGFR mutation status seemed not to be an effectively predictive marker for efficacy in patients with previously treated NSCLC. Thus more researches were still needed to identify patients who would most likely benefit from the appropriate treatment, and future focus should include identifying predictive markers which might enable treatments to be targeted to specific patient groups and thereby translate into improved outcomes.

With regard to the targeted agents used, the combined therapy of erlotinib differed between included studies, but all studies used erlotinib-based doublet therapy, and combined targeted agents included bortezomib [30], everolimus [37], bevacizumab [31], R1507 [36], tivantinib [32], sorafenib [33], sunitinib [34] or entinostat [35], respectively. Because most of these agents were novel targeted therapies that had been evaluated in phase I/II trials, limited survival and safety data was available for these novel targeted agents. Therefore, more high quality phase III RCTs were warranted to confirm the efficacy and toxicities of combining targeted therapy versus established monotherapy with erlotinib in previously treated NSCLC.

As the main aims of treatments in the metastatic setting were to prolong life, provide cancer-related symptom relief, minimize treatment-related toxicity, and improve quality of life, toxicity was particularly relevant for patients with advanced NSCLC. Finding of our study indicated that there were more incidences of grade 3 or 4 rash, fatigue, and hypertension in combining targeted therapy. With regard to the risk of grade 3 or 4 diarrhea and anemia, equivalent frequencies were found between the two groups.

Several limitations had to be mentioned in relation to this meta-analysis. Firstly, this meta-analysis was not based on individual patient data. And meta-analyses based on published data tended to overestimate treatment effects compared with individual patient data analyses. In addition, it precluded a more comprehensive analysis such as adjusting for baseline factors and other differences that existed between the trials from which the data were pooled. Therefore, the results must be interpreted cautiously, as an individual patient data-based meta-analysis would give more reliable estimation than one based on abstracted data. Secondly, we could not discover the possible survival benefits of combining targeted therapy in different NSCLC patient groups with different histologic types, detailed stages, ages, general conditions, etc., of patients, because of inadequateness of corresponding data in these eligible trials. Although all these eligible trials used erlotinib-based targeted therapy as second-line treatment for advanced NSCLC, the exact regimens among these trials were multitudinous. Thus, our study could not answer that which regimens would be the best choice. Thirdly, different treatment duration was a potential factor increasing the heterogeneity among included trials. In our study, seven included trials reported that treatment for patients was continued until disease progression, unacceptable toxicity, or withdrawal of consent, while patients in the trial conducted by Witta S.E. et al could receive up to six cycles of therapy [35]. In addition, different combining targeted therapies might also increase heterogeneity among included trials. Fourthly, combining Phase II and Phase III trials in our study was another major limitation. As OS was not always the primary endpoints for them, especially for phase II trials, therefore, the follow-up in study using tumor response as primary endpoint might be shorter than those using OS as primary endpoint, which led to a question of equivalent data maturity across studies. In our study, only three included trials used OS as the primary endpoint, thus the efficacy and safety of combining targeted therapy in advanced NSCLC patients were still needed to be investigated during the long time follow-up of these trials. Finally, in the meta-analysis of published studies, publication bias was important because trials with positive results were more likely to be published and with null results tend not to be published. Our paper observed no publication bias except for ORR and involved six studies with null results.

In conclusion, targeted therapies had revolutionized both the treatment of NSCLC as well as our understanding of the underlying molecular pathways. Although our meta-analysis demonstrated a survival benefit of combining targeted therapy over erlotinib alone as second-line treatment for advanced NSCLC, one should be cautious when interrupting these results due to the limitations of our studies. In addition, the magnitude of negative clinical studies highlights the fact these combining treatments were not “one size fits all”. Thus more studies were still needed to identify patients who will most likely benefit from the appropriate combining targeted therapy.

## Supporting Information

Checklist S1 **PRISMA checklist.**
(DOC)

Search Strategy S1 **EMBASE search strategy.**
(DOC)
