# Supplementary material for: Overall Survival Benefits for Combining Targeted Therapy as Second-Line Treatment for Advanced Non-Small-Cell-Lung Cancer: A Meta-Analysis of Published Data
Source: PLoS One. 2013 Feb 8;8(2):e55637. doi: 10.1371/journal.pone.0055637 (PMC3568141; doi:10.1371/journal.pone.0055637)
Supplement: Search Strategy S1 — EMBASE search strategy. (DOC) [file pone.0055637.s002.doc]

**EMBASE search strategy**

#1 ‘Clinical trial’/

#2 ‘Randomized controlled trial’/

#3 Randomization/

#4 Single-Blind Method/

#5 Double-Blind Method/

#6 Cross-Over Studies/

#7 ‘Random Allocation’/

#8 Placebo/

#9 Randomized controlled trial*.tw.

#10 Rct.tw.

#11 Random allocation.tw.

#12 Randomly allocated.tw.

#13 Allocated randomly.tw.

#14 (allocated adj2 random).tw.

#15 Single blind*.tw.

#16 Double blind*.tw.

#17 ((treble OR triple) adj blind*).tw.

#18 Placebo*.tw.

#19 Prospective study/

#20 OR/#1-#19

#21 Case study/

#22 Case report.tw.

#23 Abstract report/ OR letter/

#24 OR/#21-#23

#25 #20 NOT #24

#26 (Lung* OR Respiratory* OR Bronchial*).mp. [mp=title, abstract,

subject headings, heading word, drug trade name, original title, device manufacturer,

drug manufacturer name]

#27 (carcin* OR cancer* OR neoplasm* OR tumour* OR tumor* OR cyst* OR

adenocarcin* OR malign*).mp.

#28 exp Squamous/

#29 OR/#26-#28

#30 exp Adenocarcinoma/ Bronchiole alveolar carcinoma/ Large cell undifferentiated carcinoma

#31 #27 AND (#29 OR #30)

#32 metastatic/ OR advanced/

#33 previously treated/ OR second-line/

#34 targeted therapy/

#35 exp erlotinib/ OR tarceva/

#36 OR/#35-#36

#37 #32 OR #33 AND #36

#38 combine*.mp

#39 exp Combined Targeted Therapy

#40 #38 OR #39

#41 #31 AND #37 AND #25

#42 #25 AND #31 AND # 40
